# Acute Collateralization through Varicose Dilatation of the Omental Venous Arch of Barkow after Splenic Vein Collapse Induced by Pancreatitis

**DOI:** 10.5334/jbr-btr.1180

**Published:** 2017-01-26

**Authors:** Bruno Coulier, Isabelle Bueres

**Affiliations:** 1 Clinique Saint-Luc, Bouge, Belgium, BE; 2 St Luc Bouge (Namur), BE

**Keywords:** Acute pancreatitis, Greater omentum, Venous Arch of Barkow

## Case Report

**Figure 1:**
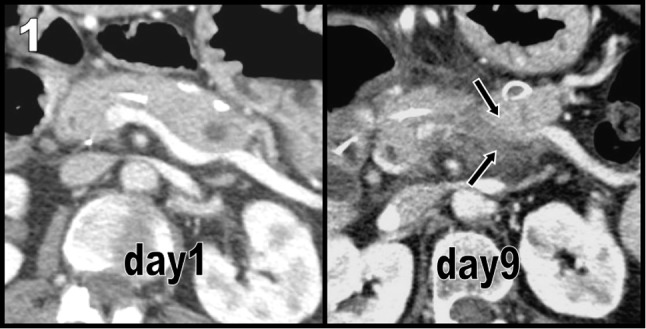


A 53-year-old female with a long history of severe episodes of acute pancreatitis was admitted with complaints of recurrent epigastric pain. Contrast-enhanced computed tomography (CT) – at day one – demonstrated only a small infracentimetric pseudocyst in the pancreatic tail, but peripancreatic effusions were absent at this time. Chronic calcified retracted sequelae of previous pseudocysts were still visible along the anterior aspect of the corporeo caudal pancreas. A stent was present in the cephalic Wirsung, and chronic collateral venous pathways had developed along the great and lesser curvatures of the stomach. The splenic vein was patent (Figure 1 left).

**Figure 2:**
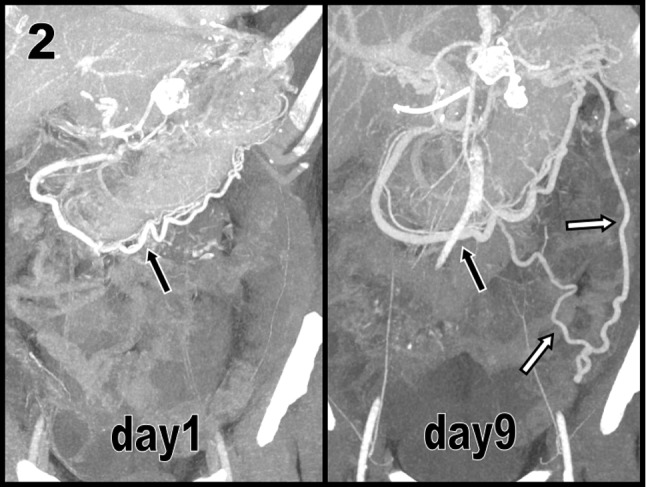


**Figure 3:**
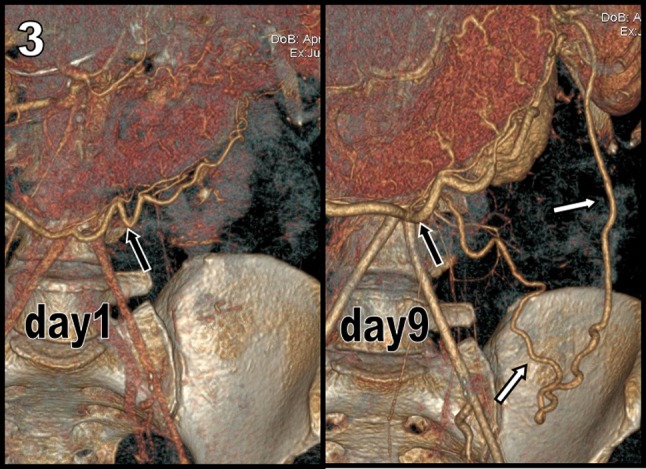


Severe recrudescence of symptoms after one week justified a new contrast-enhanced CT at day nine after admission. Effusions had now developed in the retro caudal pancreatic area, causing acute collapse of the splenic vein (black arrows on Figure 1). As a consequence, acute venous collateralization (Figure 2 maximal intensity projections, Figure 3 volume rendering views) amplified, causing an increase of the caliber of the already dilated main gastroepiploic vein (black arrow on Figures 2 and 3) and unusual varicose vein dilatation of the omental venous arch of Barkow (white arrows on Figures 2 and 3).

## Discussion

Gastric varices may classically develop as a collateral venous pathway when isolated splenic vein occlusion occurs during various diseases. Dilatation of the main gastroepiploic vein that runs along the great curvature of the stomach may also occur. Most of these different collateral pathways are spontaneously opacified during contrast-enhanced CT, particularly in patients with a long history of chronic pancreatitis or recurrent episodes of acute pancreatitis.

The speed of development of these collateral pathways remains unknown. It probably depends on the speed of obstruction of the splenic vein itself. Our case emphasizes that the development of a collateral venous pathway may be rapid, if not frankly acute.

The case also illustrates that other less traditional and less frequently described collateral pathways exist. They may be found in the transverse mesocolon – not illustrated here – and may also develop in the greater omentum, as in our case.

During embryology, the primitive omental bursa bulges downward in front of the transverse mesocolon and the transverse colon, then it adheres intimately to them, forming the greater omentum. Therefore, three distinctive mesenteric reflections exist around the pancreas: the transverse mesocolon, the gastrocolonic ligament, and, more distally, the greater omentum. The anatomic continuity of these reflections to the left is constituted by the gastrosplenic ligament and splenic hilum and to the right by the head of the pancreas. Venous collaterals may classically develop in these different mesenteric reflections when the splenic vein is occluded.

The large collateral venous arch that develops in the distal omentum and thus frequently in the lower abdomen – as reported here – represents hypertrophy of a normal venous anastomosis between the left and right epiploic veins. This anatomic arcade – or venous arch of Barkow – is the venous counterpart of the corresponding distal arterial anatomosis of the right and left descending epiploic branches of the gastroepiploic artery called the arcus epiploica magnus of Barkow.
